# An IFI6-based hydrogel promotes the healing of radiation-induced skin injury through regulation of the HSF1 activity

**DOI:** 10.1186/s12951-022-01466-x

**Published:** 2022-06-18

**Authors:** Jie Hao, Mengyi Sun, Dong Li, Tao Zhang, Jianjun Li, Daijun Zhou

**Affiliations:** 1 Department of Oncology, Southwest Hospital, Army Medical University, Chongqing, 400038 China; 2 Department of Rehabilitation, The Second Affiliated Hospital of Xinjiang Medical University, Ürümqi, 830092 China; 3 Department of Oncology, The General Hospital of Western Theater Command of PLA, Chengdu, 610083 China

**Keywords:** Polydopamine, Graphene oxide, Sodium alginate, Interferon-alpha inducible protein 6, Radiation-induced skin injury

## Abstract

Radiation-induced skin injury (RISI) is a common complication of radiotherapy. Interferon-alpha inducible protein 6 (IFI6) significantly reduces the radiation sensitivity of HaCaT cells. Sodium alginate (SA) has substantial moisturizing properties. Graphene oxide (GO) is a suitable substrate with physical antibacterial properties. Therefore, we designed materials to modify IFI6 using the biogule of polydopamine (PDA) connected to GO/SA. The structure, size, morphology, and elemental compositions of IFI6-PDA@GO/SA were analyzed. Cytological studies suggested that IFI6-PDA@GO/SA is non-toxic to HaCaT cells, with antibacterial properties. It promotes migration and vascularization and inhibits apoptosis. These cells express IFI6 after irradiation. The mouse model suggested that IFI6-PDA@GO/SA promotes wound healing and reduces reactive oxygen species expression. IFI6-PDA@GO/SA accelerates RISI healing, possibly by initiating the SSBP1/HSF1 signaling pathway. In addition, IFI6-PDA@GO/SA improves the immune microenvironment. This study constitutes the first use of IFI6 as a RISI wound-healing material.

## Introduction

Radiation resembles the standard treatment protocol for cancer types such as head and neck, breast, and lung cancers [1]. However, many complications are associated with radiotherapy reactions, limiting the radiotherapy dose and treatment effect. Radiation-induced skin injury (RISI) is a common but debilitating side effect; various degrees of radiodermatitis occur in 95% of cancer patients following radiotherapy [2]. The precise mechanisms of RISI remain unclear, and there is a lack of standardized, uniform methods to prevent and treat RISI [3]. Most hydrogels lack antibacterial and anti-reactive oxygen species (ROS) properties, leading to wound infections. Although conventional hydrogels protect wounds, the need for suture fixation to the wound (due to the lack of adhesion ability) inevitably leads to secondary damage. The defects of these hydrogels prompted us to explore a better strategy for repairing damage defects [4].

Graphene oxide (GO) is an essential material in materials science. Graphene has received substantial attention as a traumatic material [5]. These contribute to bonding through the interaction of hydrogen and π-bonding in the preparation of polymers [6]. GO materials are used in wound materials to modify their surface using non-toxic and harmless biopolymers to improve adsorption properties. Polydopamine (PDA) surfaces contain abundant catechol and amine, effective for heavy metal removal. In a weakly basic environment, self-polymerization obtains dopamine, enabling polydopamine to adsorb on the surface of almost all solid substances, forming a PDA film [7]. PDA adsorbed on the surface of materials can be reacted with agents containing nucleophilic groups by Michael addition or Schiff base reaction as a reaction “bridge,” thereby introducing other functional groups on the surface of materials [8]. Polymers modified by PDA showed good adsorption properties, antibacterial, anti-ROS, and other positive effects in wounds [9].

Interferon-alpha inducible protein 6 (IFI6) regulates apoptosis and immune responses [10]. It mediates responses to dengue and hepatitis B virus infections, reducing apoptosis of vascular endothelial cells and hepatitis B virus-specific CD8^+^ T cells, and displays antiviral activity [11]. Activating transcription factor 3 (ATF3) downregulates IFI6 to suppress the growth and migration of tongue squamous cell carcinoma cells [12]. A study of mRNA expression profiling showed that 50 upregulated and 13 downregulated genes (including IFI6) were upregulated. Overexpression of IFI6 promoted cell proliferation and reduced apoptosis and ROS production after radiation [13]. Sodium alginate (SA) (a natural polysaccharide) has been used in biomedical tissue engineering because of its adhesiveness and biocompatibility [14]. Studies showed that SA-based hydrogels induced M2 polarization of macrophages in the inflammatory phase to reduce fibrosis and scar formation during skin regeneration [15, 16].

In the present work, we prepared sprayable IFI6-PDA@GO/SA composite hydrogels. We tested their ability to promote the proliferation and migration of HaCaT cells, endowing the hydrogel with synergistic radioresistance ability in vitro and in vivo. We also examined the mutually-promoting relationship between IFI6 and PDA@GO/SA. We investigated the bioactivity of IFI6 in wound healing by measuring in vitro proliferation, migration, angiogenesis, and therapeutic efficacy against RISI in HaCaT cells, using sprayable IFI6-PDA@GO/SA hydrogels for skin regeneration. We focused on the analysis of the mechanisms of their biological activities. The sprayed IFI6-PDA@GO/SA hydrogels were simple and immediately available, making them suitable for wound treatment in emergencies such as RISI. Thus, IFI6 can be sprayed because of portability and convenience, and the dual functions of anti-ROS and skin wound healing-PDA@GO /SA hydrogels facilitated the carrier treatment of RISI patients.

## Materials and methods

### Ethics statement

Animal experiments were performed with the approval of the Animal Ethics Committee of Army Medical University. HaCat and VEGF cells were provided by the Department of Oncology, Southwest Hospital, Army Medical University. The Experimental Animal Department of the Army Medical University provided male BALB/c mice (25 g), maintained in circadian rhythm (12 h), relative humidity (50%), and ambient temperature (25 °C).

### Preparation of IFI6-PDA@GO/SA

IFI6 was obtained from SAB Co. (Maryland, USA). GO and SA were prepared by RuiXi Materials Tech Co. (Xian, China). Dopamine was obtained from Sigma-Aldrich Co. (Shanghai, China). Anti-IFI6 and anti-phospho-HSF1 were purchased from Bioss Co. (Beijing, China). Anti-SSBP1 was purchased from FineTest Co (Wuhan, China).

To synthesize PDA@GO: 30 mg GO, we added 10 mm Tris buffer (pH 8.5) and placed it in a water bath ultrasound for 2 h. We then added 30 mg DA, stirred at room temperature for 48 h, centrifuged at 8000 rpm for 10 min, washed three times with water and ethanol, and dried at 40 ℃ for 12 h.

We synthesized IFI6- PDA@GO /SA: Dissolve SA in a 100-ml beaker, then added PDA@GO, 50 µl peroxide solution, and horseradish peroxidase. Finally, we added 3 mg IFI6 protein powder into the beaker and stirred for 1 h until the mixture was a hydrogel at room temperature (25 ℃).

### Characterization

The particle size and zeta potential were measured using dynamic light scattering. Scanning electron microscopy was used to observe the morphology of the IFI6-PDA@GO/SA. Fourier transform infrared (FTIR) spectra were recorded using a Nicolet 6700 FTIR spectrometer (4000–600 cm^−1^). UV–Vis-NIR measures the UV spectrometer/near-infrared light absorption effect. Energy Dispersive Spectroscopy (EDS)/X-ray Photoelectron Spectroscopy (XPS) analyzed the elemental composition. Western blotting was used to measure the protein composition.

### Biocompatibility evaluation and cell morphology observation

Cytological experiments were divided into five groups: Group A was the normal cell group; Group B was the normal cell + 3 Gy radiation group; Group C was the normal cell + 3 Gy radiation + SA hydrogel group; Group D was the normal cell + 3 Gy radiation + PDA@GO/SA hydrogel group; Group E was the normal cells + 3 Gy radiation + IFI6-PDA@GO/SA hydrogel group. Cytotoxicity was evaluated using CCK-8 assays.

HaCat cells were seeded onto groups A–E at 2 × 10^3^ cells/well in Dulbecco's Modified Eagle Medium. Cell viability was quantified using CCK-8 assays. To study the influence of cytoskeleton morphology, HaCaT cells were seeded in media of Groups A–E for 24 h. Then, cells were incubated with 2-(4-amidinophenyl)-6-indolecarbamidine dihydrochloride (DAPI). Fluorescence images of stained cells were obtained using a confocal laser scanning microscope (780, Zeiss, Germany).

### Bacterial co-culture

Methicillin-resistant *Staphylococcus aureus* (MRSA) and *Escherichia coli* were obtained from the Army Medical University. We drew equal amounts of bacterial liquid to coat the plates. After incubation at 37 °C for 24 h, the changes in bacterial numbers were counted.

### Scratch wound migration

HaCat cells were cultured into 24-well plates (2 × 10^4^/well). Wounds were created using pipette tips drawn across the monolayers (0 h), and a Zeiss video microscope was used for inspection at 24 h. ImageJ 1.48V software (NIH, USA) was used for measurements. These assays were performed in triplicate.

### In vitro tube formation assay

Briefly, HaCaT cells were seeded into Matrigel-coated 96-well plates, followed by the addition of Groups A–E. Newly formed tubes were photographed using an inverted phase microscope (Olympus, USA). The length of the tubes and the number of tubes were measured using Image J software.

### Flow cytometry

According to the manufacturer's instructions, apoptosis was measured using a flow cytometry assay and an Annexin V-FITC/PI Apoptosis Detection kit (Dojindo Molecular Technologies, Japan). Briefly, cells were collected and suspended in 200 µl of binding buffer containing 5 µl of Annexin V-FITC and 5 µl of propidium iodide. Then, the cells were incubated at room temperature for 15 min in the dark. CD4^+^, CD8^+^, NK cells, and M1 cells in the RISI wound's immune microenvironment were counted using flow cytometry. The cells were analyzed using Cell Quest software using a Fluorescence Activated Cell Sorter (Beckman Coulter, USA).

### Cell clone formation assay

Cell suspensions from groups A–E were added to the low-concentration agarose solution described above to achieve a final concentration of 1000/ml. Each well was irradiated at 3 Gy. Each well was stained with 0.005% crystal violet (1 ml) for 1 h, and the number of colonies was counted using Image Pro Plus 6.0 after imaging.

### The RISI mouse model

Each mouse was anesthetized by intraperitoneal injection with approximately 0.2 ml pentobarbital. The linear accelerator emits 6 MeV electron rays (one irradiation; 30 Gy; irradiation field 1 cm × 1 cm; dose 300 cGy/min; 10 min). The source skin distance was 1 m, and the remaining skin was blocked using a lead plate. After each group was irradiated, the irradiated part was covered with material and replaced every other day for 7–14 days after irradiation and divided into five groups, with five mice in each group. Cytological experiments were divided into five groups as described above.

The wound areas before and after healing were compared to calculate the healing rate using IPP6.0 software. The wound-healing rate = (the initial wound area – the wound area after healing for a specific time)/the initial wound area × 100%. RISI was scored according to the method of Douglas and Fowler.

### Hematoxylin–eosin (HE) staining and histological analysis

Wound samples from each mouse were taken 14 days after wounding to produce paraffin sections for HE staining. High-quality images were selected. Several pathology experts measured the length of the neoepithelium in a blinded fashion.

### Western blot and immunohistochemical staining detection of SBPP1/HSF1 expression

Fourteen days after the injury, 10 mm × 10 mm samples were taken from the full-thickness defect, including the neoepidermis and granulation tissue, and immediately frozen in liquid nitrogen according to the manufacturer's instructions (Varioskan Flash; Thermo Scientific, USA). Anti-IFI6 (Biuss, China), anti-sbpp1 (Finetest, China), and anti-hsf1 (Biuss) were diluted 1:100. Goat anti-rabbit secondary antibody labeled with goat horseradish peroxidase (Zhongshan Bio, China) was diluted to 1:2000. Polyvinylidene fluoride membranes were harvested and sent for chemiluminescence observation (Thermal Scientific, USA). For immunohistochemistry, the steps were identical.

### Quantitative real-time PCR and ROS measurements

We performed real-time PCR of NLRP3 on the 7500 Real-Time PCR System (Applied Biosystems Instruments), using SYBR Green Master Mix (TOYOBO, QPK-201). Enzyme-linked immunosorbent assay kits were used to measure ROS changes in each group.

### Statistical analysis

Each experiment was performed in triplicate, and all data were expressed as mean ± standard error of the mean. Two groups were compared using the Student’s t-test, and differences were considered statistically significant at P < 0.05.

## Results and discussion

### Fabrication of IFI6- PDA@GO/SA

**Fig. 1:**
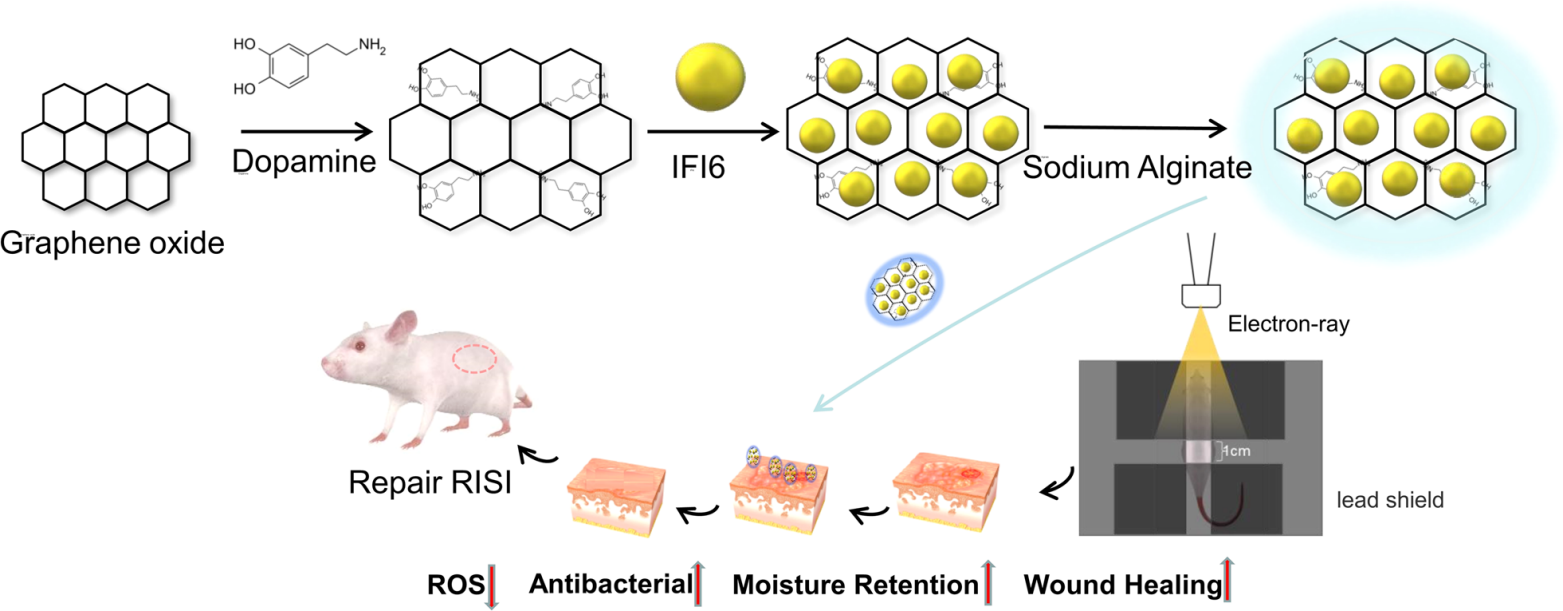
Schematic illustration showing the fabrication of IFI6-PDA@GO/SA and its application for RISI healing

To meet the needs of RISI wounds and the complex process of wound healing, we prepared a multifunctional composite nanomaterial with excellent reduced radiosensitivity, moisture retention, biological safety, anti-ROS, and anti-hypoxia capabilities. The fabrication process of IFI6-PDA@GO/SA is shown in Fig. 1.

### Characterization of IFI6-PDA@GO/SA

**Fig. 2:**
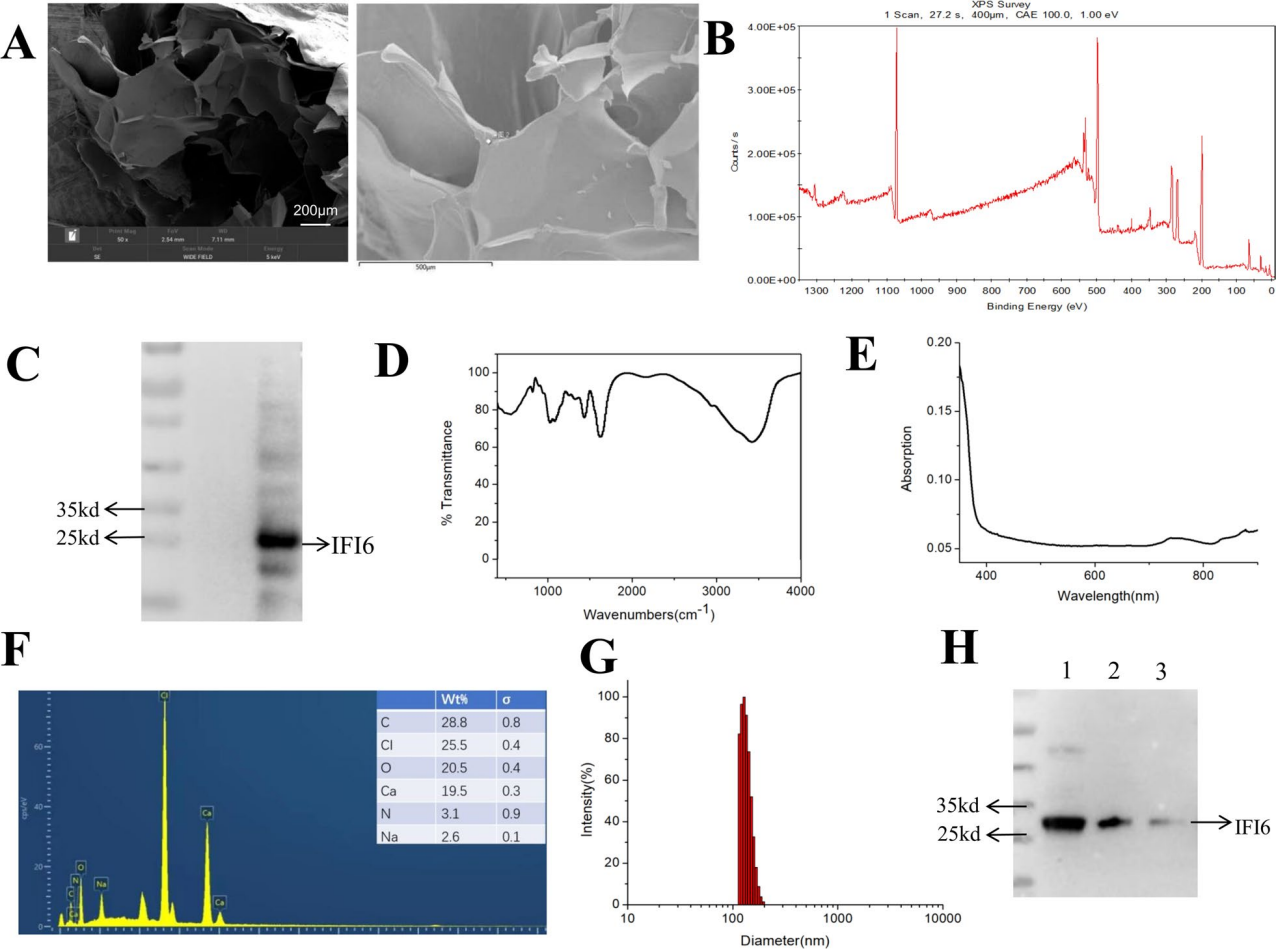
Characterizations of IFI6-PDA@GO/SA. **A** SEM (left) and EDS (right) images of IFI6-PDA@GO/SA. **B** XPS of IFI6-PDA@GO/SA. **C** Gel electrophoresis results of IFI6-PDA@GO/SA. **D** FTIR of IFI6-PDA@GO/SA. **E** The ultraviolet spectrum of IFI6-PDA@GO/SA. **F** EDS of IFI6-PDA@GO/SA. Test elements include O, N, Na, CI, Ca, C. **G** Particle size and potential analysis. D = 162 nm, PDI = 0.19, zeta potential = − 14.64 mV. **H** SDS–PAGE protein analysis of native IFI6 (1), IFI6 released from the IFI6-PDA@GO/SA after incubated with (2) or without (3) GSH, respectively, for 48 h

IFI6- PDA@GO /SA material is semi-solid. Scanning electron microscopy (SEM) showed that the sodium alginate substrate looked like a sheet-like structure. Ma et al. reported that SEM images showed that the SA hydrogels have pore structures on the micrometer scale [17]. The addition of β-FeSi_2_ did not affect its morphology. Higher magnification EDS images showed that IFI6- PDA@GO The particles were distributed on the inner side of the pore wall (Fig. 2A).

The element mapping images of EDS and XPS survey indicated that the elements O, N, Na, CI, Ca, and C were uniformly distributed in IFI6-PDA@GO/SA (Fig. 2B, F). C, N, and O were identified in IFI6, C and O presented in SA, and Na, CI, and Ca appeared in PDA@GO. These findings suggest that IFI6, SA, and PDA@GO were found in the composite sponge. GO sheets were obtained using a modified Hummer’s method with an average size of 2 μm measured using Image J software and a thickness of about 1.5 nm measured by Atomic Force Microscope [18]. To characterize the chemical composition of the Fe_3_O_4_NPs-GO-PDA composite thin film, Jang et al. suggested that the C peak (284.08 eV), the peak of C−C (284.08 eV), and the peak of O 1s (531.95 eV) were remarkably dominant, suggesting that the composite system consisted of GO sheets; C−O and C−N binding energies were observed at 286.56 and 285.79 eV, respectively, suggesting that the PDA contains C−N bonds [19].

Liu et al. found characteristic absorption bands of GO sheets at 1054 cm^−1^ (alkoxy), 1224 cm^−1^ (epoxy), 1401 cm^−1^ (carboxyl; C–O), and 1724 cm^−1^ (carboxyl; C=O) [18]. The new adsorption peak at 1579 cm^−1^ of the MPDA@GO composites may be attributed to the deformation vibration of N–H bonding and the stretching vibration of C–N bonding. The FTIR results in this paper are consistent with these findings (Fig. 2D), suggesting that the reaction may have occurred between epoxide groups of GO and amine groups of PDA during the preparation of PDA@GO composites. There are no reports of the characteristic absorption peak of IFI6. However, we believe that the 3400 cm^−1^ position may be associated with the composition of IFI6.

To further test the loading of IFI6, sodium dodecyl sulfate–polyacrylamide gel electrophoresis (SDS-PAGE) analysis was conducted (Fig. 2C). We found that the IFI6- PDA@GO/SA material expressed IFI6, consistent with the IFI6 protein bands as reported [13], suggesting that IFI6 was successfully tested. IFI6-PDA@GO/SA displays a typical nanostructure, and the diameter is about 162 nm, PDI = 0.19, and zeta potential = − 14.64 mV (Fig. 2G). The ultraviolet spectrum of IFI6-PDA@GO/SA showed no evident change in 400–800 nm (Fig. 2E). Zhao et al. synthesized polyelectrolyte complex nanoparticles for plasmid delivery [20]. Nanosized SA solids ranged from 0.04% to 0.08%, and higher concentrations resulted in larger agglomerate-like solution systems. Active targeting of small particles has advantages over passive targeting of large nanospheres due to the enhanced permeability and retention effect. Xie et al. measured zeta potential and found that the SA sponge was negatively charged; the zeta potential of the composite sponges was dominated by the SA ratio [21]. The antioxidant glutathione (GSH) degrades GO [22]. To demonstrate the stability of IFI6 protein in our nanocarriers and the degradability of the nanocarriers, the degradation products of IFI6-PDA@GO/SA after incubation with GSH for 48 h were collected and subjected to SDS-PAGE. Figure 2H shows SDS-PAGE images of native IFI6 and IFI6 released by IFI6-PDA@GO/SA with and without GSH treatment for 48 h. Compared with the characteristic band of native IFI6 (lane 1), IFI6-PDA@GO/SA without GSH treatment (lane 3) showed a very light color band, while IFI6-PDA@GO/SA with GSH treatment showed a band similar to native IFI6 (lane 2).

### Biocompatibility and antibacterial activity of IFI6-PDA@GO/SA

**Fig. 3:**
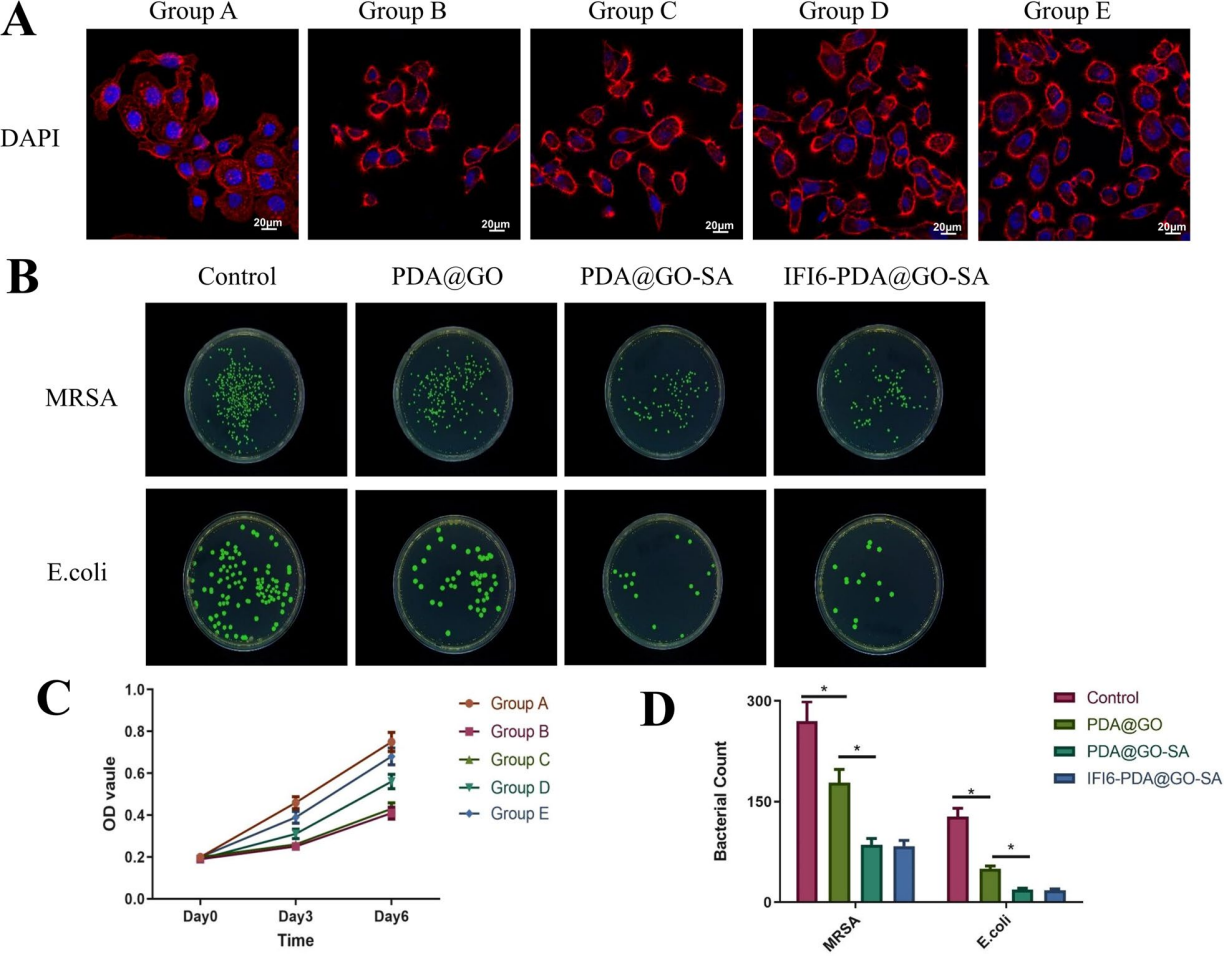
Biocompatibility and antibacterial activity of IFI6-PDA@GO/SA. **A** FITC/DAPI staining images of HaCaT cells cultured on IFI6-PDA@GO/SA on day 7. **B** Antimicrobial activity of G + (*MRSA*) and G– (*E. coli*) with IFI6-PDA@GO/SA. **C** CCK-8 assay of HaCaT cells cultured on IFI6-PDA@GO/SA on day 7. **D** Bacterial count of B. *P < 0.05

HaCaT cells and IFI6-PDA@GO/SA were co-cultured for 7 days, and FITC/DAPI staining showed that the cell morphology was not significantly different (Fig. 3A). The nucleus and cytoplasm were intact. The CCK-8 assay on day 3 showed that the optical density of the HaCaT cells in Groups B and E was reduced after 4-Gy irradiation, suggesting that radiation significantly inhibited cell growth (Fig. 3B). On day 6, the optical density of Group E recovered significantly and was significantly higher than that of Group B (P < 0.05), suggesting that IFI6-PDA@GO/SA does not affect the growth of irradiated cells in the short term (1–3 days), while the growth curve of irradiated cells returns to normal in the long term (> 6 days). Jia et al. used an EdU incorporation assay to measure cell proliferation ability and found that IFI6 overexpression in HaCaT and WS1 cells restored the significant increase in cell proliferation after radiation [13]. In contrast, the downregulation of IFI6 resulted in less cell proliferation than in the control group. Liu et al. measured the cell viability properties of the PDA@GO hydrogel [18]. After 5 days of incubation, cell viability improved in all three samples. Although studies reported that GO has some toxicity, the composite hydrogel exhibits good biocompatibility without any toxic effects of graphene oxide. These findings suggest that the SA hydrogels prevented the toxicity of GO in the PDA@GO/SA composite hydrogels. Furthermore, PDA and SA are biodegradable, whereas GO is nonbiodegradable, suggesting that this composite hydrogel might be suitable for RISI treatment.

Bacterial infection of skin wounds delays healing and may even cause wound deterioration. Improving the antibacterial performance is critical to developing novel hydrogel wound dressings; to this end, various antibacterial agents have been introduced into the hydrogels. Huang et al. reported that the hydrogels containing PDA@Ag5GO1 (Ag5GO1 denotes that the mass ratio between Ag and GO is 5:1) exhibited effective antibacterial properties and high inhibition of *E. coli* and *S. aureus* [23]. Liu et al. showed that CNF hydrogels and MPDA@GO (1:2)/CNF composite hydrogels showed no antibacterial effect against *E. coli* and *S. aureus* [18]. The (MPDA-TH)@GO (1:2)/CNF composite hydrogel inhibited *E. coli* growth with a zone width of 15 mm, whereas the drug-loaded composite hydrogel exhibited a zone width of 18 mm against *S. aureus*. This finding suggests that the inhibition zone of the (MPDA-TH)@GO (1:2)/CNF composite hydrogel depends on the released TH from the composite hydrogel. MRSA (G^+^) and *E. coli* (G^−^) in the control group grew well; however, PDA@GO inhibited growth (Fig. 3B, D). PDA@GO /SA inhibited bacterial growth to a greater extent, and IFI6- PDA@GO /SA did not show sufficient bacteriostasis. These findings may be related to the fact that IFI6 has no reported antibacterial activity, while GO and SA have been reported to have good antibacterial activity. 

### In vitro cytological study of IFI6-PDA@GO/SA

**Fig. 4:**
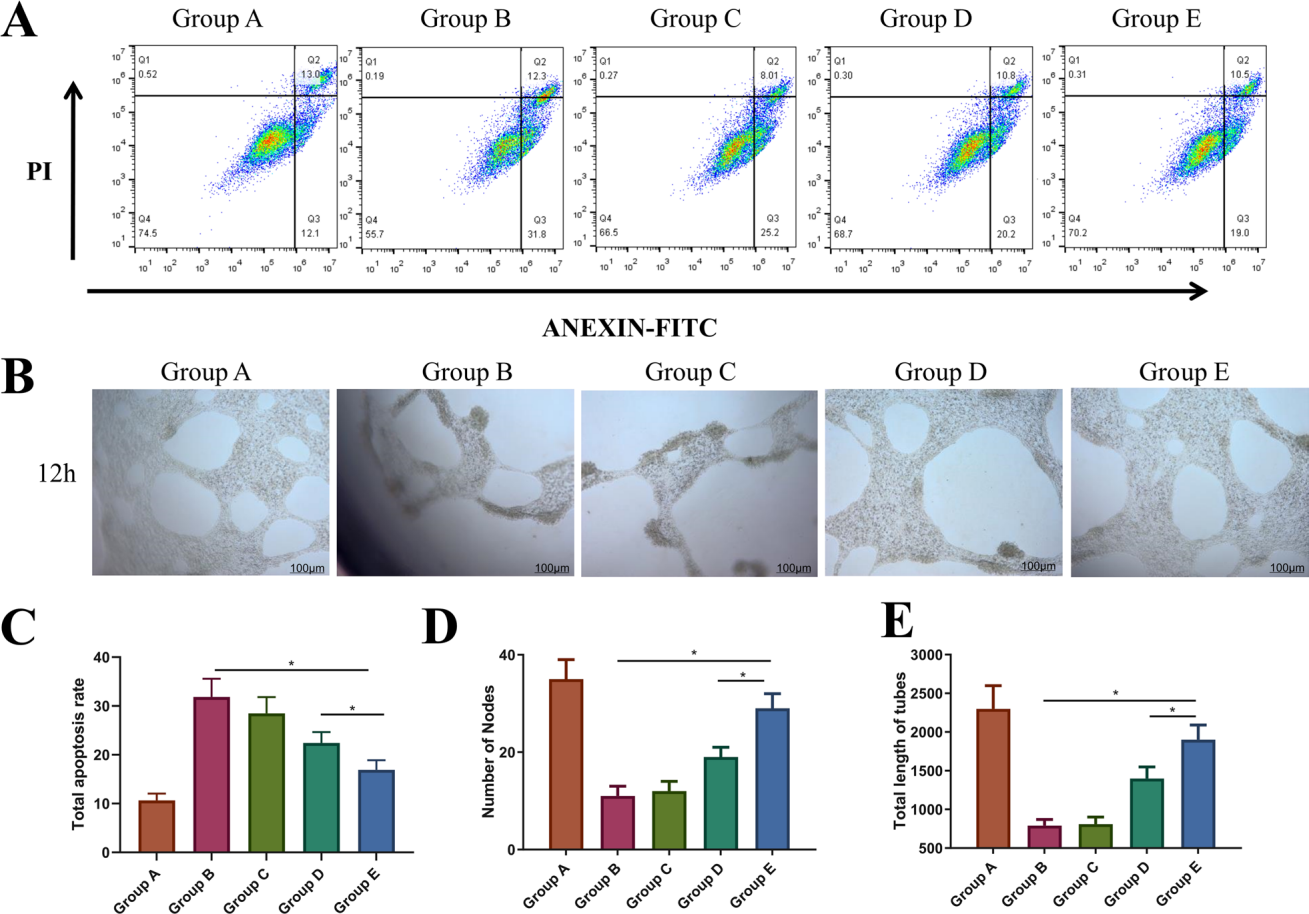
The in vitro cytological study of IFI6-PDA@GO/SA. **A** + **C** Flow cytometry and its total apoptosis rate results. **B** In vitro tube formation assay. **D**, **E** The number of nodes and total length of tubes in Group B. *P < 0.05

We performed flow cytometry assays to determine the potential role of IFI6 in apoptosis. IFI6 is only expressed in higher eukaryotes [12]. Radiation significantly increased the apoptosis rate (Fig. 4A, C). Compared with Group B, Group E had a lower apoptosis rate (P < 0.05), and the effect in Group E was higher than that of Group D. This finding suggests that IFI6-PDA@GO/SA significantly reduces apoptosis and that the increased IFI6 protein in Group E participates in regulating apoptosis. These phenomena may be related to anti-ROS mechanisms.

IFI6 overexpression resulted in decreased apoptosis in irradiated HaCaT and WS1 cells compared with control skin cells, suggesting a survival-promoting function of IFI6. The effect of IFI6 on mitochondrial membrane potential was investigated (an essential indicator of mitochondrial function) using JC-1 staining [13]. After irradiation, the authors showed that IFI6-silenced WS1 cells had decreased mitochondrial membrane potential. IFI6 regulated melanoma development and growth through E2F2-mediated DNA replication [24] and promoted breast cancer cell metastasis by inducing deregulation of the mitochondrial redox state [25]. In the present study, we showed that overexpression of IFI6 in HaCaT cells promoted cell survival through anti-apoptosis and guided local aggregation of surviving cells by promoting cell migration, suggesting a new function of IFI6 promoting tumor cell survival. Yin et al. found that overexpression of CTD-3252C9.4 facilitated apoptosis of pancreatic cancer cells in vitro and in vivo [10]. IFI6 overexpression counteracted the effects of CTD-3252C9.4 upregulation on the survival and apoptosis of pancreatic cancer cells. These findings suggest that preventing the transcription of IFI6 might restrain tumor vascularization and cell migration. Zhang et al. found that the survival of 3T3 fibroblasts on Ag-PDA/BC(rGO) composite films was > 90% at 24 h [9]. This phenomenon may be related to Ag or BC components, but not PDA and RGO. Nevertheless, we believe that PDA and RGO have no inhibitory effect on HaCaT cells.

As shown in Fig. 4B, D, E, after co-cultivating vascular endothelial cells with materials of different groups, the Matrigel angiogenesis experiment was carried out for about 24 h. The number of blood vessels of normal cells in Group A was the highest, and the blood vessels were the longest. The formation of blood vessels was significantly restricted after 4-Gy irradiation in Group B, while the miR181a@RBC-HB precursor material in Group D partially restored blood vessel formation (P < 0.05); Group E contained IFI6 material. The best pro-angiogenesis effect of serotonin was in Group E (P < 0.05). Another study found that overexpression of IFI6 counteracted the inhibition of TSCC cell growth and migration induced by the overexpression of activating transcription factor 3 (ATF3) [12]. IFI6 loss inhibited esophageal squamous cell carcinoma progression through ROS accumulation caused by mitochondrial dysfunction and ER stress. This phenomenon may be associated with promoting vascularization by IFI6 [26].

Recent findings demonstrated that IFI6 is an interferon-stimulated gene enriched in the inner mitochondrial membrane; it is a proliferative and anti-apoptotic factor [25]. IFI6 may promote breast cancer metastasis by regulating mitochondrial ROS production. Liu et al. examined the abundance of IFI6 in esophageal squamous cell carcinoma tissues [26]. IFI6 promoted ESCC cell proliferation and survival by regulating redox homeostasis.

### In vitro cytological study of IFI6-PDA@GO/SA

**Fig. 5:**
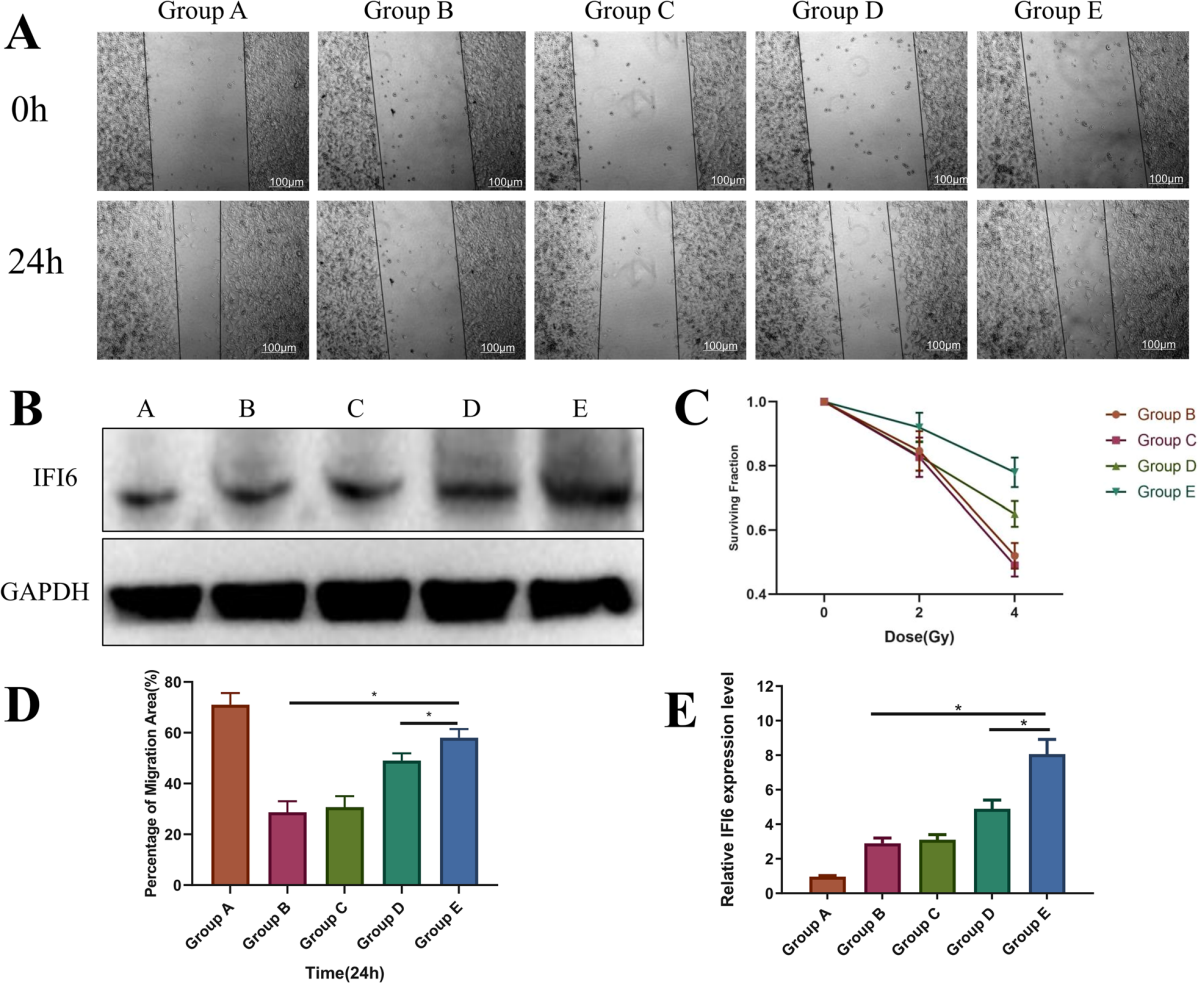
The in vitro cytological study of IFI6-PDA@GO/SA. **A** Scratch wound migration of HaCaT cells with IFI6-PDA@GO/SA. **B** + **E** Western Blot of IFI6 and Its semi-quantitative analysis. **C** Cell clone formation assay. **D** Percentage of migration area of Group A. *P < 0.05

As shown in Fig. 5A + D, HaCaT cells were co-cultured with materials in the various groups for about 24 h and then subjected to cell scratch tests. The 24-h migration rate of Group B was significantly lower (P < 0.05), possibly related to the effect of radiation on cell migration. The migration inhibition of Group E improved significantly, suggesting that this material can increase the migration rate of irradiated cells (P < 0.05). It is worth noting that the cell migration rate of Group E was higher than that of Group D (P < 0.05), suggesting that increased IFI6 protein expression promotes the migration of HaCaT cells. IFI6 exerts critical anti-viral and anti-apoptosis functions; however, its role in ionizing radiation-induced stress of skin cells has not been reported. Western blotting demonstrated that the successful overexpression of IFI6 significantly facilitated the proliferation rate after 5-Gy X-radiation [13]. Consequently, apoptosis and ROS generation of IFI6 overexpressing B16 F10 cells significantly decreased. These results suggest that IFI6 confers radioresistance in cancer cells, suggesting a novel radiotherapy target. IFI6 expression in the western blotting analysis is shown in Fig. 5B, E. The expression of IFI6 in Group E was significantly higher than that of Groups B and D (P < 0.05), suggesting that the material may enter the cytoplasm of HaCaT cells through endocytosis, thereby exerting related effects.

To confirm the protective effect of IFI6 protein on HaCaT cells against radiation, we adopted the “gold standard” paradigm. A cell clone formation experiment showed that, after 4-Gy irradiation, the relative clone number of the simple irradiation group (Group B) decreased by 45% compared with before irradiation (P < 0.05). In comparison, the relative clone number of Group E only decreased by about 20% (P < 0.05) (Fig. 5C). This finding suggests that IFI6 protein enhances the anti-radiation ability of cells, which is conducive to the formation of cell clones.

### The effect of IFI6-PDA@GO/SA on the RISI mouse model

**Fig. 6:**
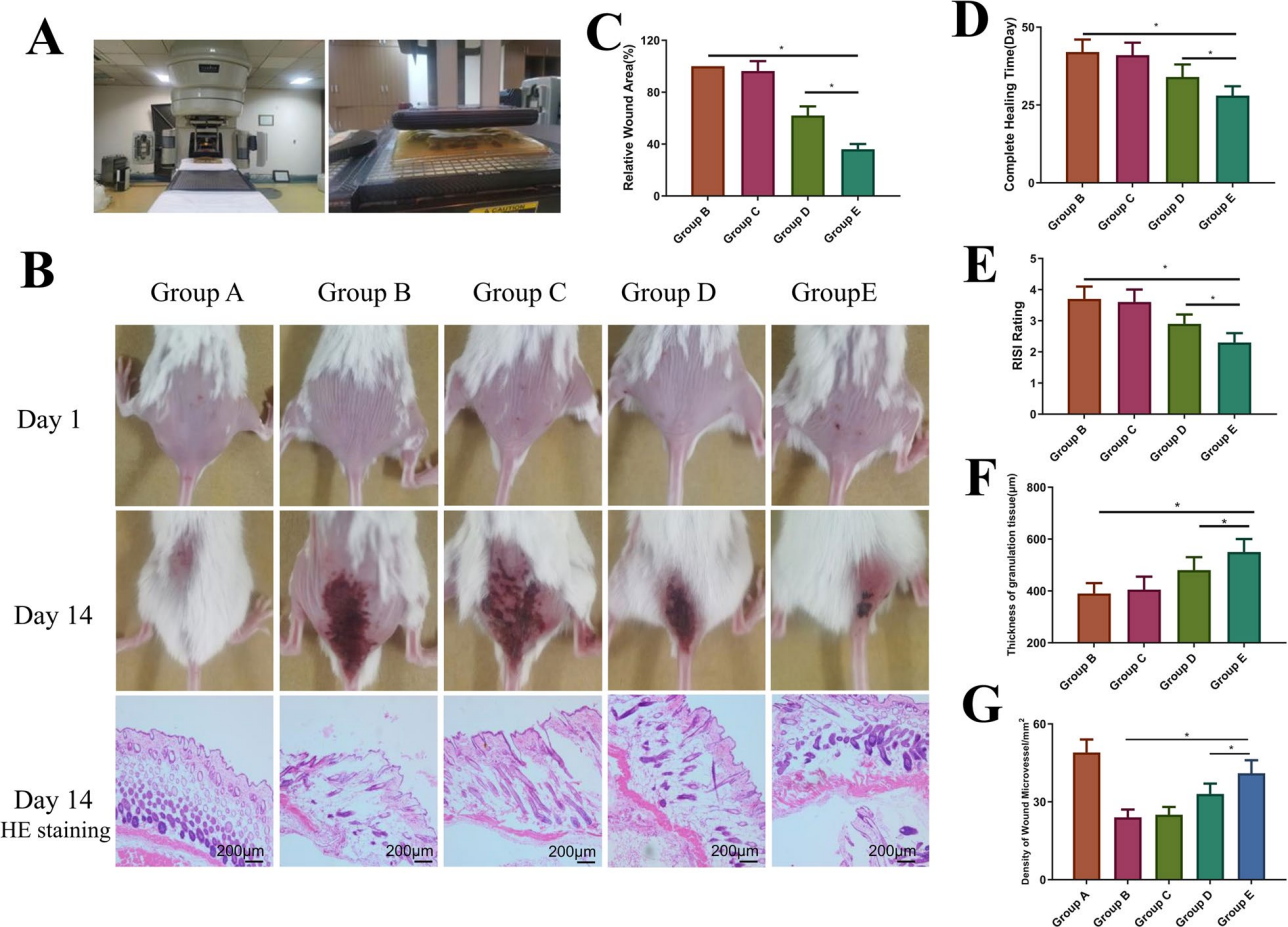
The in vivo mouse study of IFI6-PDA@GO/SA. **A** Medical electron linear accelerator and operation process in the mouse RISI model. **B** Photographs of wounds on days 1 and 14 and HE staining on day 14. **C** Wound area for each group on day 14. **D** Complete healing time. **E** RISI rating on day 14. **F**, **G** The thickness of granulation tissue and density of wound microvessels on day 14. *P < 0.05

In Fig. 6A, the picture on the left shows the electronic beam radiotherapy equipment, and the picture on the right shows the positioning and modeling process of the mouse animal model. As shown in Fig. 6B, all mice were shaved and imaged to establish a model treated on day 1. On day 14, the mice were photographed again. These mice were sacrificed, and local skin tissues were collected for HE staining (Fig. 6C). Compared with Group B, Groups D and E promoted wound healing (P < 0.05), and the effect of Group E was significantly higher than that of Group D (P < 0.05), suggesting that IFI6 promoted the migration and proliferation of epidermal cells, thereby promoting wound healing. As shown in Fig. 6A, inflammation was significantly observed in all groups on day 14. The epidermis formed in Group E. Some gene-based drugs were shown to treat RISI diseases; however, they were unstable in vivo, limiting their application. For example, fibroblast growth factor-2 heals RISI damage; however, free FGF-2 is sensitive to proteolytic enzymes and heat [27]. Therefore, a sufficient concentration of gene drugs must be maintained locally to sustain release while retaining activity. Nanomaterials with good drug-loading capacity can address these issues. In the present study, we enhanced the efficacy of IFI6 to form the nanocomplex IFI6-PDA@GO/SA, which was delivered into the skin by subcutaneous administration before X-irradiation [27]. This design significantly increased the stability of IFI6 and stabilized, activated, and released IFI6 from IFI6-PDA@GO/SA.

This nanosystem possesses the following advantages: (i) as a biological glue, PDA has a significant anti-ROS effect and biocompatibility. GO is characterized by a large specific surface area, rich functional groups, easy modification, and antibacterial properties. Thus, the encapsulation of PDA@GO facilitates permeation into the stratum corneum and delivery of IFI6 into the skin; (ii) IFI6 promotes cell proliferation and migration in RISI wounds. Moreover, IFI6 showed a good anti-apoptotic effect; (iii) SA provides moisture retention, and the wet environment is conducive to wound repair of RISI. These findings suggest the potential application of the nano-transfersomes in defending against RISI. As shown in Fig. 6D, in terms of total healing time, mice in the simple irradiation group (Group B) completely healed for about 44 days, consistent with the current mainstream view. miR181a@EM-HB significantly reduced healing time to 31 days (P < 0.05). The healing effect was the strongest in Group E, with a complete healing time of about 26 days, significantly better than in Group D (P < 0.05).

Radiation Therapy Oncology Group (RTOG) scores were used to evaluate RISI mice. This study used RTOG scores to evaluate wound healing at 14 days subjectively. Groups B and C scores were identical, and the wounds reached RTOG 3–4, except for skin folds, primarily fusion, and wet desquamation/pitting edema; some even showed ulceration, hemorrhage, and necrosis (Fig. 6E). The wounds of group D were significantly improved, RTOG 2–3, showing patchy wetness desquamation/moderate edema. The subjective degree of the wounds in group E was the best (RTOG grade 2), and the primary manifestations were fresh and bright erythema.

There have been studies on biomaterials for RISI. Kyritsi et al. designed a nonwoven patch composed of electrospinning polymerized micro/nanofibers loaded with an aqueous extract of pine halepensis bark and clinically tested its efficacy in preventing radiation dermatitis [28]. No adverse events were reported, suggesting that the patch might be a safe medical device for prophylactic radiation dermatitis treatment.

During wound repair, the number and quality of blood vessels directly affect wound healing [29]. Analysis of the HE staining (Fig. 6B) showed that the granulation tissue thickness (Fig. 6F) and wound blood vessel density (Fig. 6G) were essentially identical. Radiation reduces granulation tissue thickness and blood vessel density, leading to wounds. New epithelial growth and wound vascularization processes are inhibited, delaying wound healing. IFI6-PDA@GO/SA significantly increased the thickness of wound granulation tissue and increased the density of wound blood vessels, promoting wound healing and improving radiation inhibition. group E contains IFI6 protein, suggesting its most potent promoting effect (P < 0.05). Figure 6G displays the quantitative statistics for IFI6-PDA@GO/SA, suggesting that nanomaterial promoted wound healing by improving angiogenesis. Zhao et al. studied fullerenol, known as a “free radical sponge” [2]; the authors found that fullerenol significantly blocked ROS-induced damage and improved the viability of irradiated human keratinocytes. In vivo experiments showed that medical sodium hyaluronate hydrogels containing fullerenol were suitable for dermal administration, protected epidermal stem cells, and alleviated radiation dermatitis.

### Mechanism of IFI6-PDA@GO/SA

Upregulated expression or activation of the NLRP3 inflammasome plays a critical role in RISI [30]. Extremely high medium wave UV irradiation causes an inflammatory reaction in the skin. Hasegawa et al. found that UVB-induced activation of the NLRP3 inflammasome promotes IL-1β and secretion of other inflammatory mediators, including TNF-alpha, IL-6, IL-1-alpha, and prostaglandin E2 [31]. Feldmeyer et al. showed that human keratinocytes persistently express inflammasome proteins, IL-1-alpha, IL-1-beta, and IL-18 [32]. The intracellular free Ca2^+^ level increased after UVB irradiation, causing NLRP3 inflammasome activation. Ahmad et al. demonstrated that disturbance of Ca^2+^ homeostasis leads to NLRP3 inflammasome activation in response to UVB exposure [30]. Modulation of NLRP3 inflammasome associated targets may lead to novel preventive and therapeutic strategies for RISI treatment.

**Fig. 7:**
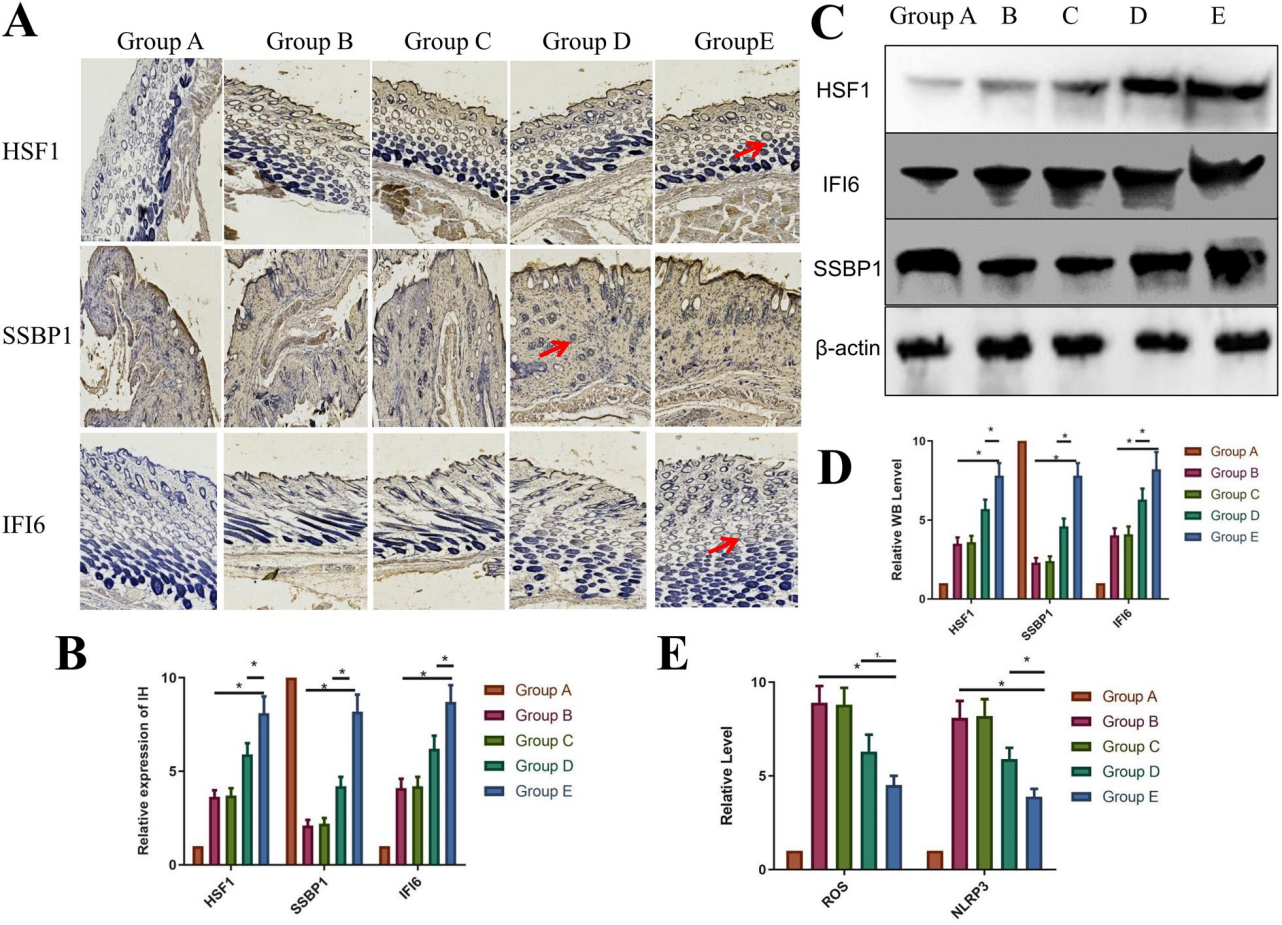
The mechanism of IFI6-PDA@GO/SA. **A** Immunohistochemistry of mouse RISI on day 14. Positive antibody expression (red arrow). **B** The relative expression of HSF1, SSBP1, and IFI6 IHC on day 14. **C**, **D** Western blot of HSF1, SSBP1, and IFI6. **E** Relative levels of ROS/NLRP3 on day 14. *P < 0.05

We performed a preliminary study of the material mechanism to promote wound healing in mice (Fig. 7). Mechanistic studies found that the expression of ROS/NLRP3 was associated with RISI [33, 34]. As shown in Fig. 7C, ROS/NLRP3 mRNA expression increased significantly after 30 Gy radiation; however, IFI6-PDA@GO/SA (Group E) reduced ROS expression; that is, from the perspective of classical pathways, IFI6 protein significantly reduces the expression of NLRP3 inflammasome. We qualitatively and semi-quantitatively analyzed the protein expression of IFI6-related pathways. Ionizing radiation-induced ROS are mediators of DNA damage [33]. The DCFH-DA fluorescent probe detected that ROS production was significantly reduced in skin cells overexpressing IFI6 after radiation exposure; conversely, IFI6 knockdown increased radiation-induced intracellular ROS levels [35].

SSBP1 is a mitochondrial housekeeping gene involved in mitochondrial biogenesis; it is a subunit of a single-stranded DNA binding complex involved in maintaining genome stability. After radiation exposure, we observed colocalization of IFI6 and SSBP1 using immunohistochemistry (Fig. 7), suggesting a potential interaction between IFI6 and SSBP1 under radiation induction. In the heat shock response (HSR), SSBP 1 relocates to the nucleus by interacting with heat shock transcription factor 1 (HSF1) [36]. Jia et al. proposed that IFI6 is involved in HSF1 mediated HSR [13]. As expected, radiation enhanced HSF1 transcriptional activity was blocked in IFI6 knockout cells but enhanced in IFI6-overexpressing cells. HSF1-mediated HSR is a typical anti-toxic stress response, including heat shock and oxidative stress.

As shown in the immunohistochemistry studies, Group A showed less expression of IFI6 and its downstream pathway SSBP1/HSF1 protein, and the positive expression of related proteins (Group B) increased significantly after 30 Gy radiation (Fig. 7A, B). IFI6-PDA@GO/SA (Group E) had the most potent effect of promoting protein expression (P < 0.05). As shown in Fig. 7C, D, the IFI6 protein in wound tissue significantly increased, suggesting that IFI6-PDA@GO/SA material released IFI6 into the wound. IFI6 co-localizes with SSBP1 to initiate the expression of HSF1, thereby mediating the downstream HSR. Elevated expression of heat shock proteins regulated by HSF1 is radioprotective for tumor cells [36]. HSF1 targets genes such as ppl to interact with AKT1, a kinase that mediates a variety of cell growth and survival signal transduction processes [13]. These states illustrate the complex mechanisms by which IFI6 regulates the radiation sensitivity of human skin cells.

### The effect of IFI6-PDA@GO/SA on the immune microenvironment

Immune cells, particularly regulatory T (Treg) cells, play a critical role in wound healing. When wound formation and inflammatory responses occur, immune cells help clear foreign antigens [37]. Treg cells suppress the activation of the immune system and prevent pathological self-reactivity such as autoimmune diseases. Cytokines participate in cell–cell interactions and communication and are involved in cell migration, proliferation, and inflammatory responses. Kim et al. found that CD4^+^ and CD8^+^ Treg cells in cm had a variety of cytokines and growth factors [38]. CD4^+^ and CD8^+^ Treg cells stimulated HaCaT keratinocyte migration through EMT and upregulation of MMP-1.

**Fig. 8:**
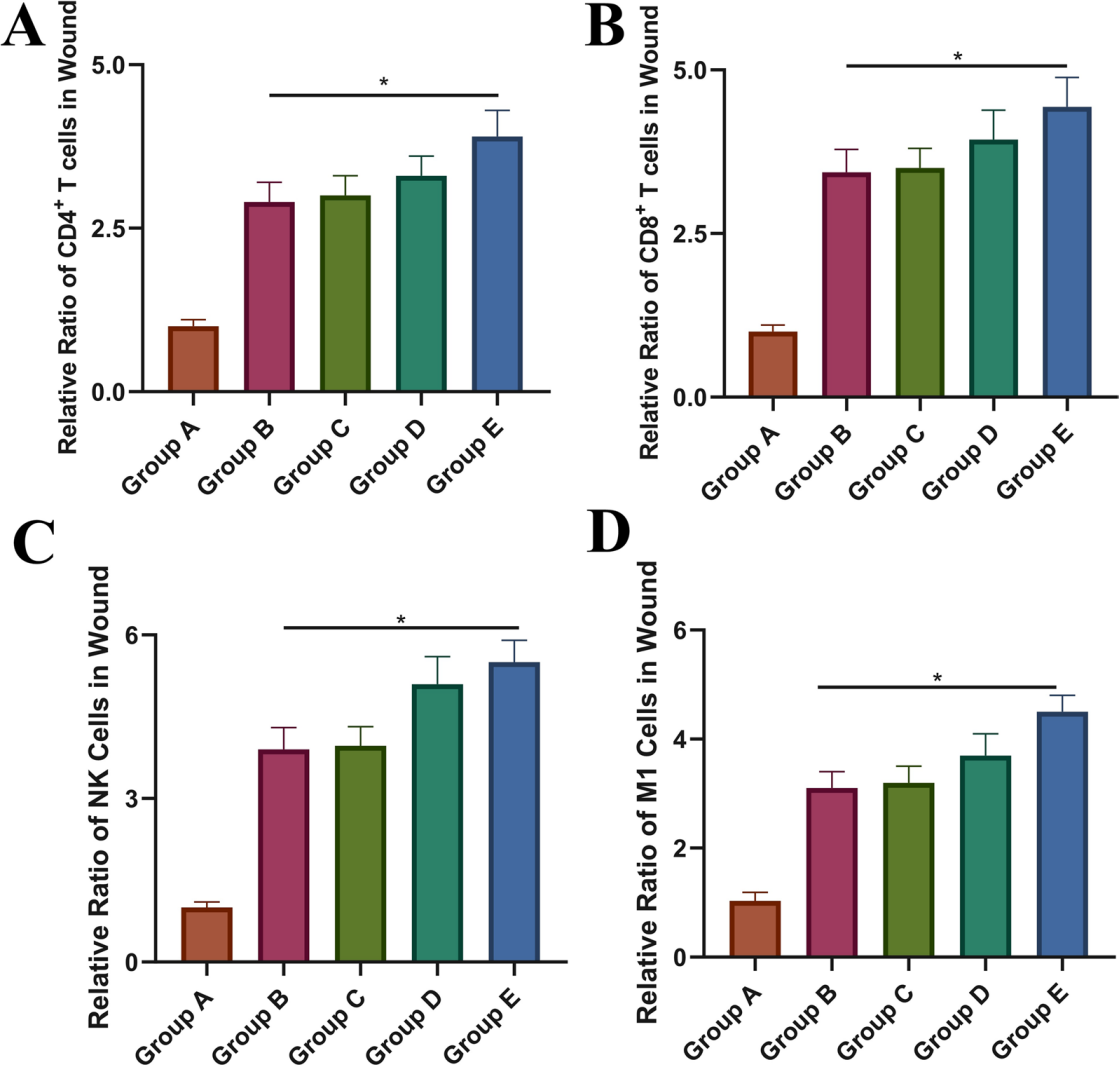
The effect of IFI6-PDA@GO/SA on the immune microenvironment. **A** Percentages of CD4^+^ T cells. **B** Percentages of CD8^+^ T cells. **C** Cells were gated on the CD45^+^ population and then on CD49b^+^ to identify NK cells. **D** Cells were further gated on F4/80^+^ CD86hi to identify M1 macrophages. *P < 0.05

To determine whether the IFI6-PDA@GO/SA regulates the immune microenvironment within the wound and explore the mechanism, wound-draining lymph nodes were collected 14 days after irradiation. Flow cytometry showed that Group E had significant activation of CD4^+^ and CD8 ^+^T cells (Fig. 8A, B). These findings suggest that Treg cells which contains various cytokines and growth factors, stimulates cell migration and proliferation to promote wound healing. IFI6-PDA@GO/SA also promoted the infiltration of NK and M1 cells (Fig. 8C, D). Sobecki et al. demonstrated that the lack of hypoxia-inducible factor (HIF)-1α in NK cell α hypomorphic mice exhibit the cytokine interferon-γ and impaired release of granulocyte–macrophage colony-stimulating factor as part of a blunted immune response [39]. HIF-1 in NK cells α is the link that balances antimicrobial skin defenses and overall repair.

It is worth highlighting that we found that IFI6-PDA@GO/SA elevated NK cells more significantly than CD4^+^ and CD8^+^ cells. On the other hand, IFI6-PDA@GO/SA promoted CD4^+^ and CD8^+^ expression in wound cells, thereby increasing T cell activation and NK cell infiltration, realizing the synergistic effect of reducing sensitization in RISI.

## Conclusions

We designed and fabricated a nanomaterial combining IFI6 for RISI wound healing. Based on our overall evaluation of IFI6-PDA@GO/SA nanomaterials, we conclude that GO possesses excellent antibacterial activity, infrastructure, and biocompatibility. PDA possesses excellent anti-ROS and bioadhesion properties. SA possesses excellent antibacterial activity and moisture retention. The most important finding is that IFI6 promotes cell proliferation, migration, and vascularization and stimulates the immune microenvironment. The five components work together to promote RISI healing. IFI6 acts synergistically to reduce oxidative stress and inflammation by activating the SSBP1/HSF1 signaling pathway.

IFI6-PDA@GO/SA improves inflammation in RISI wounds and induces granulation tissue formation, angiogenesis, and collagen deposition, resulting in faster wound closure. We believe this nanomaterial provides a valuable alternative and promising strategy for RISI wound repair.
